# Supplementary material for: Mapping the broadband circular dichroism of copolymer films with supramolecular chirality in time and space
Source: Nat Commun. 2022 Jan 11;13:210. doi: 10.1038/s41467-021-27886-1 (PMC8752614; doi:10.1038/s41467-021-27886-1)
Supplement: Supplementary file 1 — Supplementary Information [file 41467_2021_27886_MOESM1_ESM.pdf]

Supplementary Information for

Mapping the broadband circular dichroism of copolymer films with supramolecular chirality in time and space

Marius Morgenroth[1], Mirko Scholz[1], Min Ju Cho[2], Dong Hoon Choi[2], Kawon Oum[1*] & Thomas Lenzer[1*]

[1] University of Siegen, Faculty IV: School of Science and Technology, Department Chemistry and Biology, Physical Chemistry 2, Adolf-Reichwein-Str. 2, 57068 Siegen, Germany
E-mail: oum@chemie.uni-siegen.de, lenzer@chemie.uni-siegen.de

[2] Department of Chemistry, Research Institute for Natural Sciences, Korea University, 145 Anam-ro, Seongbuk-gu, Seoul 02841, Republic of Korea

Inventory of Supplementary Information

Supplementary Note 1. CD imaging of c-PFBT thin films with diffraction-limited resolution S2
Supplementary Note 2. Invariance of CD images under sample rotation and flipping S3
Supplementary Note 3. Comparison of CD images and crossed-polariser images....................S5
Supplementary Note 4. Contour diagrams of transient CD and absorption experiments S6
Supplementary Note 5. Additional UV–Vis broadband transient absorption experiments S7
Supplementary Note 6. Transient fluorescence experiments S9
Supplementary Note 7. Kinetic model S10
Supplementary Note 8. Summary of optimised fit parameters for the TrCD kinetics.............S13
Supplementary Note 9. Kinetic simulations for two limiting cases S14
Supplementary References S17

Supplementary Note 1.

CD imaging of c-PFBT thin films with diffraction-limited resolution

Supplementary Fig. 1 shows CD imaging results for four different areas (80 × 60 μm^2) of the c-PFBT thin film investigated in Fig. 2 of the main manuscript. The CD images in panels a-d look very similar, as do the corresponding CD spectra integrated over the entire field of view (210 × 160 μm^2) on the right side. The corresponding g_{abs} images are presented in panels e-h. The four regions have a very similar appearance, suggesting a good uniformity of the c-PFBT thin film over larger length scales. This is confirmed by the g_{abs} statistics on the right side of panels e-h, where the g_{abs} distributions are very similar and perfectly described by Gaussian functions with large average g_{abs} values of –0.28 ± 0.06. Note that the images in panels c and g correspond to the representative regions selected in panels a and b of Fig. 2 in the main manuscript, respectively.

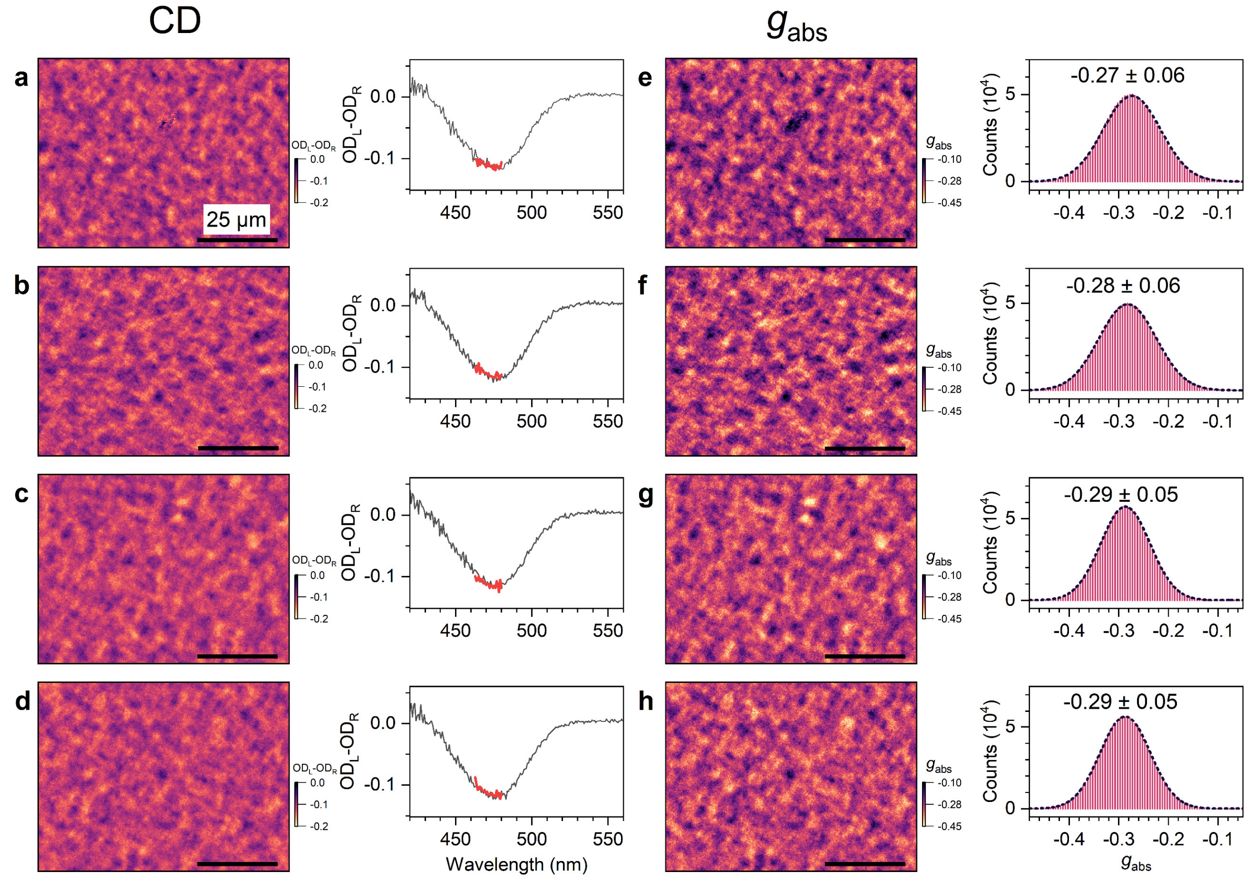

Supplementary Fig. 1 CD imaging with diffraction-limited resolution for different regions of the c-PFBT thin film analysed in Fig. 2 (main manuscript). a-d Microscope images (80 × 60 μm^2) for the circular dichroism of the c-PFBT thin film at different positions. Plots on the right show the CD signals integrated over the entire field of view (210 × 160 μm^2, thin black lines: full spectra, thick red lines: spectral region selected by the band pass filter (470 ± 10 nm) used for CD imaging). **e-h** Corresponding images for the dissymmetry factor g_{abs}, with histograms on the right including Gaussian fits as dashed black lines, determined over the entire field of view. The length of the black scale bar in each image indicates a distance of 25 μm. The images in panels c and g correspond to the panels a and b of Fig. 2 in the main manuscript, respectively.

Supplementary Note 2.
Invariance of CD images under sample rotation and flipping

Figure 1c in the main manuscript already showed that the thin films exhibit virtually no changes in the CD response and the dissymmetry factor g_{abs} upon turning or flipping of the thin film samples on a several millimetre length scale. Here, we additionally demonstrate that this also holds over the micrometre length scale observed in the CD images. An example is shown in Supplementary Fig. 2 for the same film used in Fig. 1c of the main manuscript. We intentionally picked a scratched area, which makes the 90° stepwise rotation in anticlockwise direction easily visible. The scratches also provide a convenient reference for comparing individual features in the images. As one can clearly see, the images are indeed invariant under rotation, with an average g_{abs} value of –0.32. The area-integrated spectra for OD_L, OD_R, OD_L–OD_R and g_{abs} are also virtually identical. Therefore, we can also exclude contributions on the micrometre scale resulting from a combination of linear dichroism and linear birefringence in our sample in combination with any possible anisotropies of the CD microscopy setup. As a nice side information further illustrating the power of CD imaging, we can see that in the region of the scratches there is strongly reduced CD activity (blueish colours), which indicates that the film in the scratched regions must be either very thin (low CD activity) or even completely missing.

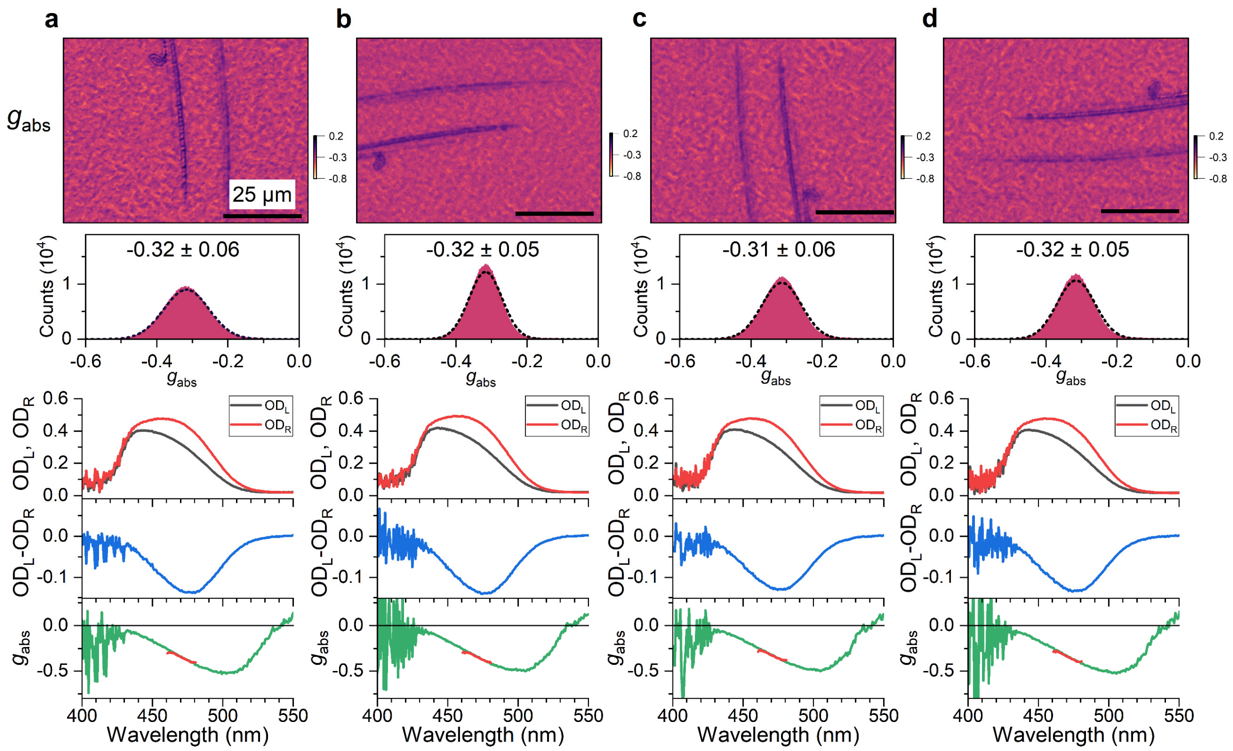

Supplementary Fig. 2 Invariance of a CD image under anticlockwise rotation of the film sample. a Microscope image (80 × 60 μm^2) for the dissymmetry parameter g_{abs} of a 240 nm thick c-PFBT thin film (top) with a histogram of g_{abs} values (middle) including a Gaussian fit (dashed black line), determined over the entire field of view, and spectra integrated over the entire field of view (210 × 160 μm^2, bottom) showing the optical density for left- and right-circularly polarised light (OD_L (black line), OD_R (red line)), the CD spectrum (blue line) and the g_{abs} spectrum (green line), with the thick red line indicating the spectral region selected by the band pass filter (470 ± 10 nm) used for CD imaging. **b-d** Same as in panel a, but sample manually rotated anticlockwise in 90° steps. The length of the black scale bar in each CD image indicates a distance of 25 µm.

We also repeated the same measurements after flipping the sample. Here we needed to search for a different scratched spot, and the results are shown in Supplementary Fig. 3. Again, the images are virtually invariant under 90° anticlockwise rotation. The average g_{abs} value of this different region is slightly lower (–0.26) but still very close to the value for the region shown in Supplementary Fig. 2. Also here, we can exclude contributions on the micrometre scale resulting from a combination of linear dichroism and linear birefringence in our sample in combination with any possible anisotropies of the CD microscopy setup.

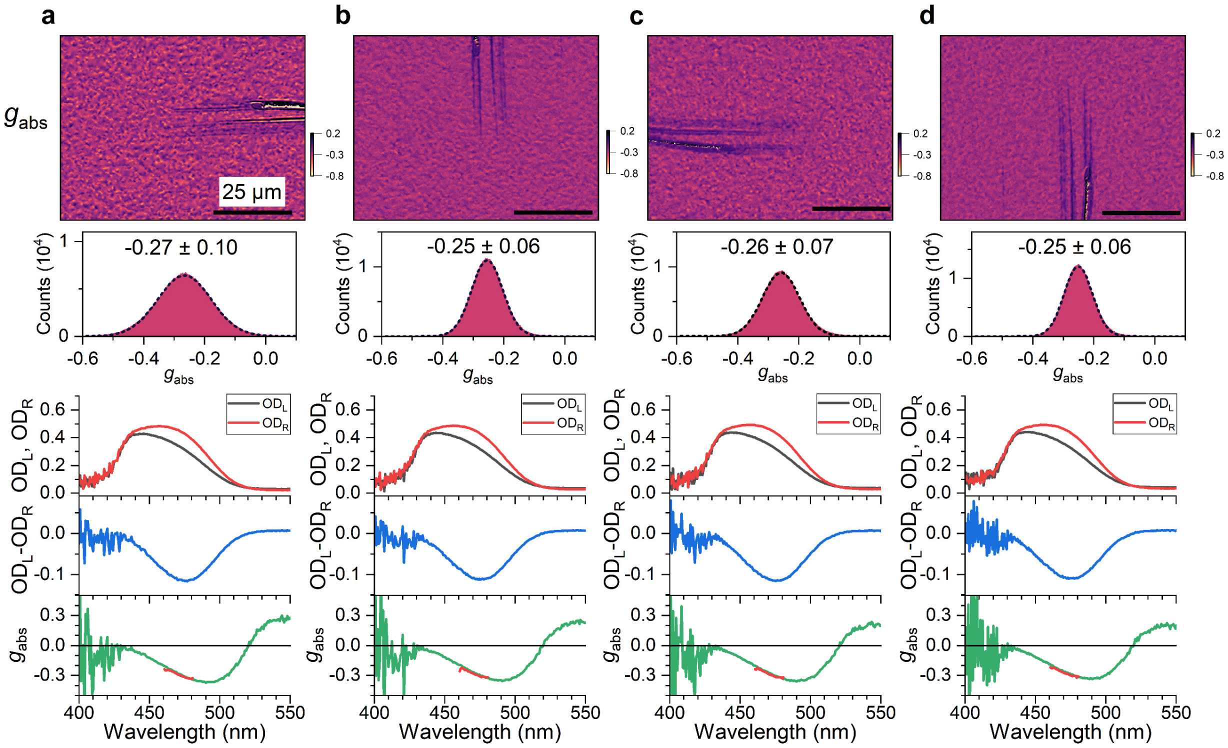

Supplementary Fig. 3 Invariance of the CD image under anticlockwise rotation after flipping the same sample used in Supplementary Fig. 2. a-d Microscope images for g_{abs}, g_{abs} distributions and absorption, CD and g_{abs} spectra, obtained using the same procedure as in Supplementary Fig. 2, employing subsequent 90° anticlockwise rotation steps.

Supplementary Note 3.
Comparison of CD images and crossed-polariser images

Supplementary Fig. 4 shows a comparison of images of a c-PFBT thin film for the dissymmetry parameter g_{abs} (panels in a, same as in Supplementary Fig. 3a-d) and images for another region of the same film using a conventional crossed-polariser arrangement (panels in b). In both cases, the images were rotated anticlockwise in 90° steps. The crossed-polariser images show very similar island-type structures which lead to changes in the polarisation of the linearly polarised illumination beam. The distinct black lines of the scratches suggest that the c-PFBT film in these regions is either very thin or missing, so that no change in polarisation takes place.

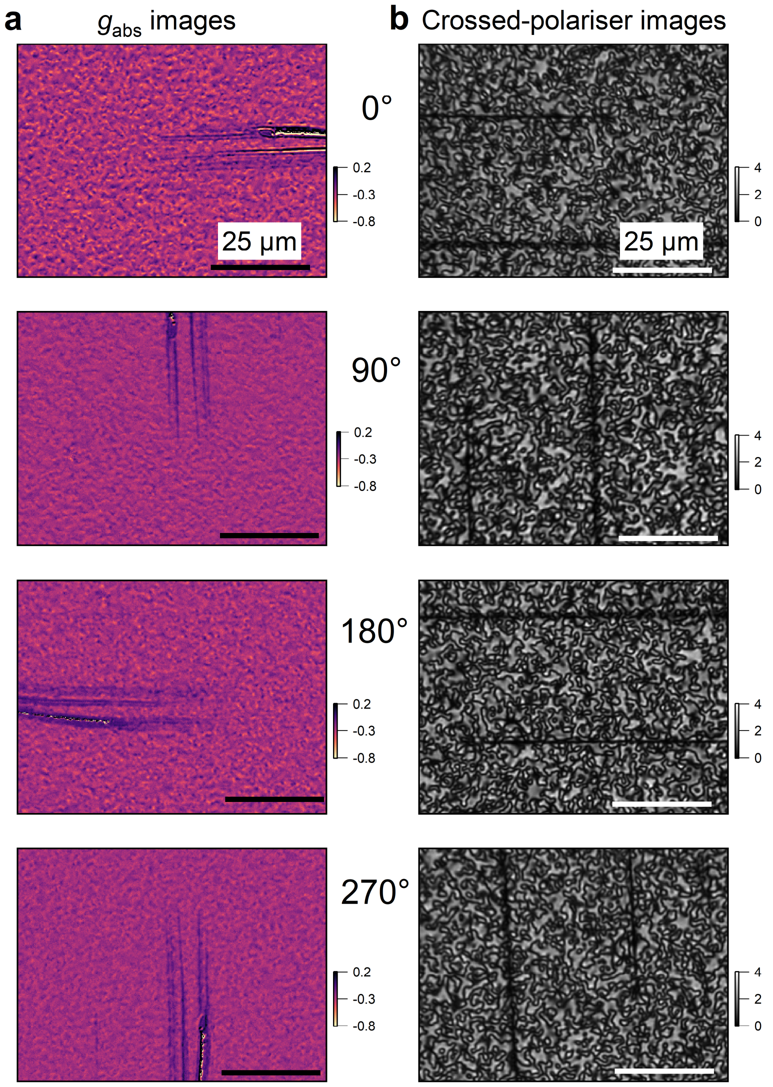

Supplementary Fig. 4 Comparison of CD images and crossed-polariser images obtained for anticlockwise rotation of a c-PFBT thin film sample. a Same microscope images (80 × 60 μm^2) for the dissymmetry parameter g_{abs} of a 240 nm thick c-PFBT thin film as shown in Supplementary Fig. 3 manually rotated in steps of 90° (from top to bottom). **b** Same as in panel a, but for another region of the same film located between crossed polarisers. The length of the black and white scale bars in each of the images indicates a distance of 25 µm.

Supplementary Note 4.
Contour diagrams of transient CD and absorption experiments

Supplementary Fig. 5 shows the complete data set of the transient absorption spectra for left-circularly and right-circularly polarized probing (panels a and b) and the resulting transient circular dichroism spectra (panel c) for the 241 nm thin c-PFBT film as contour plots. The sample was excited at 320 nm. Spectra for selected delay times from this data set were already provided in Fig. 4a of the main manuscript. It is clearly visible that the bleach regions (blue) around 340 and 455 nm of the transient absorption spectra for RCP probing (panel b) are more negative than for LCP probing (panel a). This is the reason for the positive features (red) in the TrCD contour plot (panel c), which is the difference between the contour plots in panels a and b. The TrCD spectra closely resemble the inverted steady-state CD spectrum. Weak circularly polarised stimulated emission is visible in panel c as a feature of violet colour above 520 nm.

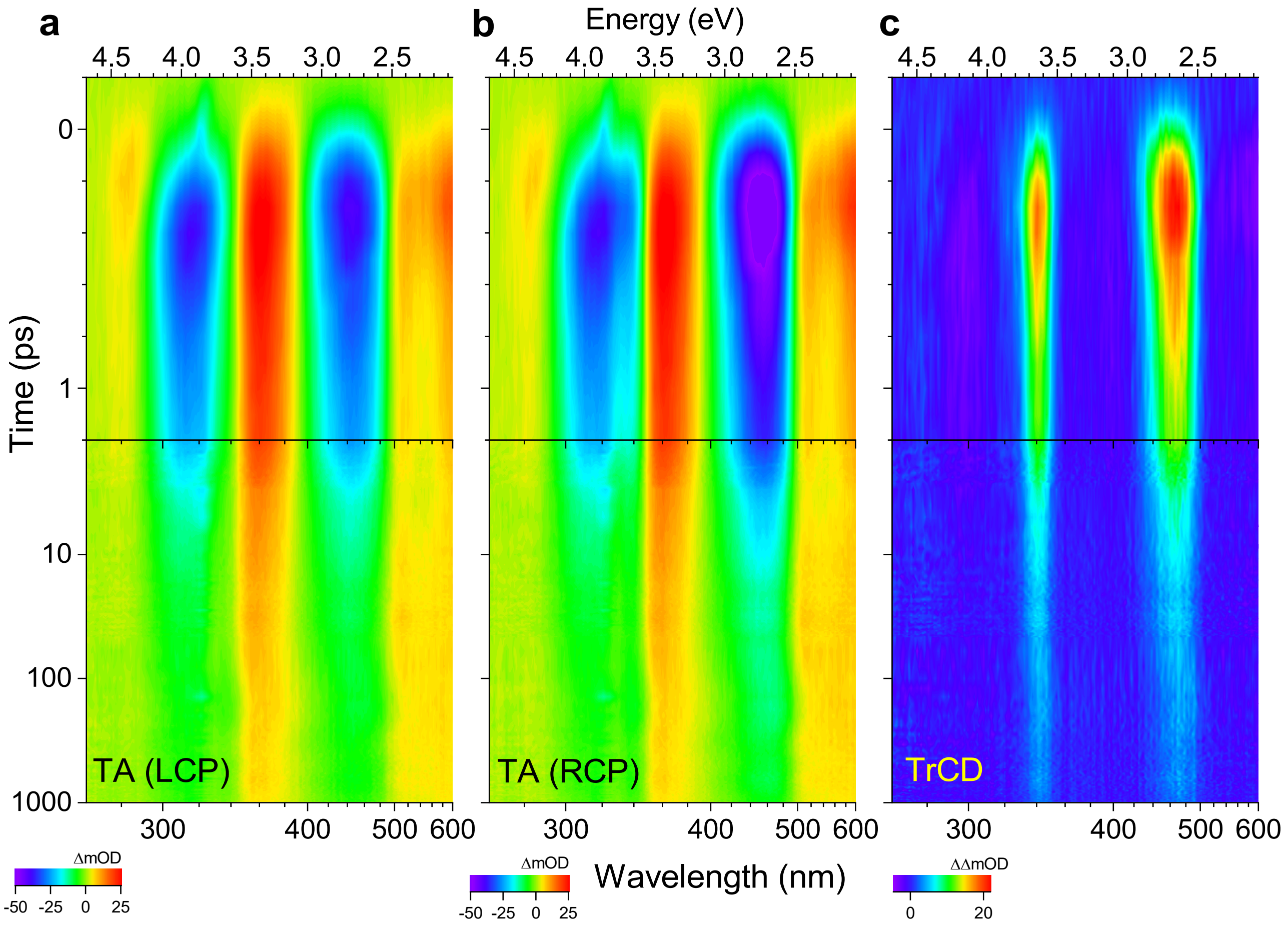

Supplementary Fig. 5 Contour diagrams of ultrafast transient absorption and transient circular dichroism spectra of a 241 nm thin c-PFBT film after excitation at 320 nm. a Transient absorption for probing with left-circularly polarised light. **b** Transient absorption for probing with right-circularly polarised light. **c** Transient circular dichroism (difference of the contour diagrams shown in panels a and b). Note the logarithmic time axis.

Supplementary Note 5.
Additional UV–Vis broadband transient absorption experiments

Supplementary Fig. 6 shows ultrafast broadband transient absorption experiments for a 157 nm thin c-PFBT film at the pump wavelengths 320 nm (S_x excitation, panels a and b) and 450 nm (S_1 excitation, panels c and d). In each case, experiments were carried out at low and high initial exciton number densities: for 320 nm excitation $N_0(S_x) = 1.5 \times 10^{18}$ and 8.9×10^{18} cm^{-3}, and for 320 nm excitation $N_0(S_1) = 1.7 \times 10^{18}$ and 1.0×10^{19} cm^{-3}.

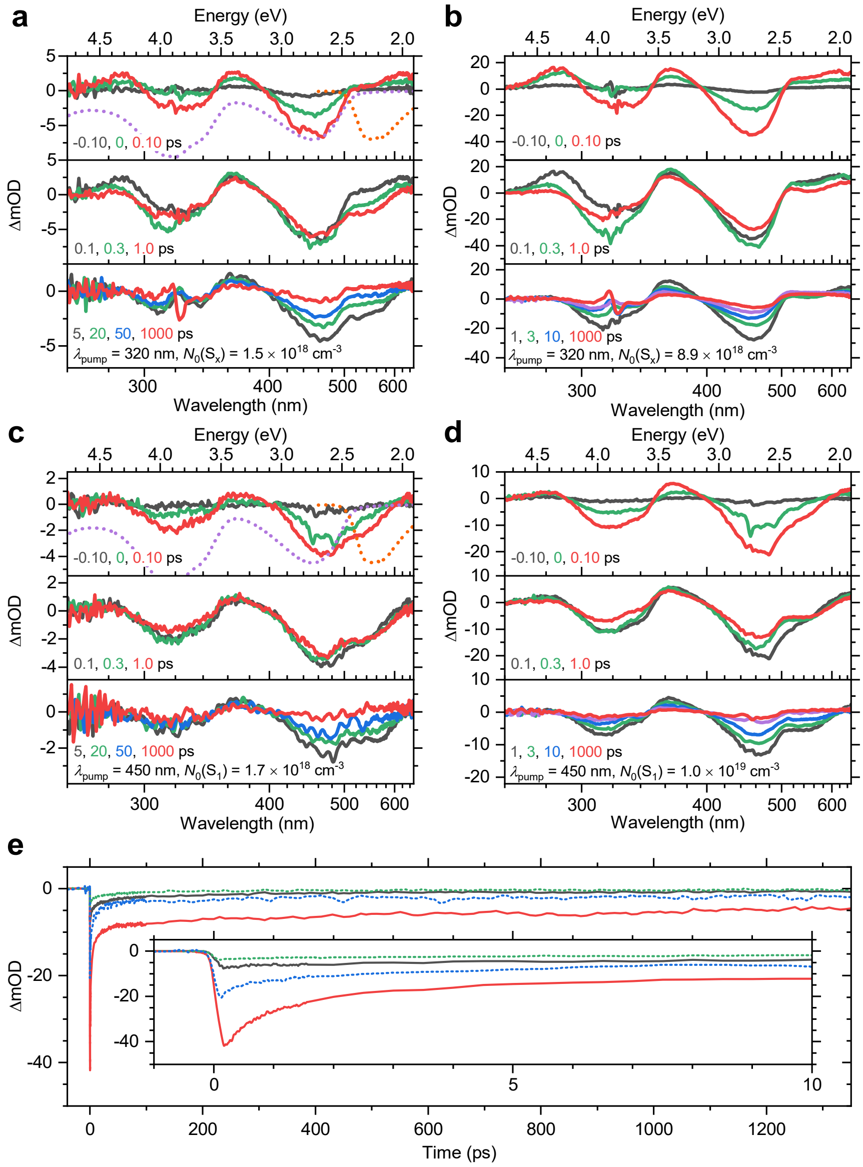

Supplementary Fig. 6 Ultrafast broadband transient absorption experiments for a 157 nm thin c-PFBT film. **a** Pump wavelength: 320 nm, $N_0(S_x) = 1.5 \times 10^{18}$ cm^{-3}. **b** Pump wavelength: 320 nm, $N_0(S_x) = 8.9 \times 10^{18}$ cm^{-3}. **c** Pump wavelength: 450 nm, $N_0(S_1) = 1.7 \times 10^{18}$ cm^{-3}. **d** Pump wavelength: 450 nm, $N_0(S_1) = 1.0 \times 10^{19}$ cm^{-3}. Dotted violet and orange lines in panels a and c are the inverted steady-state absorption and the S_1 stimulated emission spectrum, respectively. **e** Kinetics averaged over the wavelength range 460–470 nm for 320 nm excitation (black solid line: $N_0(S_x) = 1.5 \times 10^{18}$ cm^{-3}, red solid line: $N_0(S_x) = 8.9 \times 10^{18}$ cm^{-3}) and 450 nm excitation (green dashed line: $N_0(S_1) = 1.7 \times 10^{18}$ cm^{-3}, blue dashed line: $N_0(S_1) = 1.0 \times 10^{19}$ cm^{-3}), with a magnification for short times in the inset.

We focus here on additional spectroscopic information of central importance for the kinetic modelling procedure. First of all, we compare the different spectral evolution for excitation at 320 and 450 nm. Around zero time-delay, the transient absorption spectra for 320 nm excitation (panel a) show a bleach feature, which has the same shape as the inverted steady-state absorption spectrum (dotted violet line). In contrast, for 450 nm excitation (panel c) the transient spectra clearly show also a stimulated emission feature above 500 nm, which comes from the S_1 exciton state. For excitation at 320 nm (panel a), this S_1 stimulated feature is initially missing and only builds up at later times, see the spectra at 0.1, 0.3 and 1 ps. We therefore assign this process to internal conversion from the S_x state to the S_1 state. The kinetic fit in the wavelength range 525–570 nm (Supplementary Fig. 7) provides the lifetime of the S_x state as $\tau_{x,total}$ = 206 fs. As shown in the kinetic modelling (main manuscript), the corresponding rate constant $k_{x,total} = \tau_{x,total}^{-1} = 4.85 \times 10^{12}$ s^{-1} is the sum of the rate constant k_x for internal conversion from S_1 to S_x and the rate constant k_{CPx} for charge-pair formation (i.e. generation of an electron–hole pair). This value was employed as an important constraint in the kinetic modelling procedure and leads to an accurate value of the S_1–CP branching ratio. The long-lived absorption of the CP state is clearly visible in the transient absorption spectra above 600 nm (cf. the red spectra at 1000 ps in panels a–d).

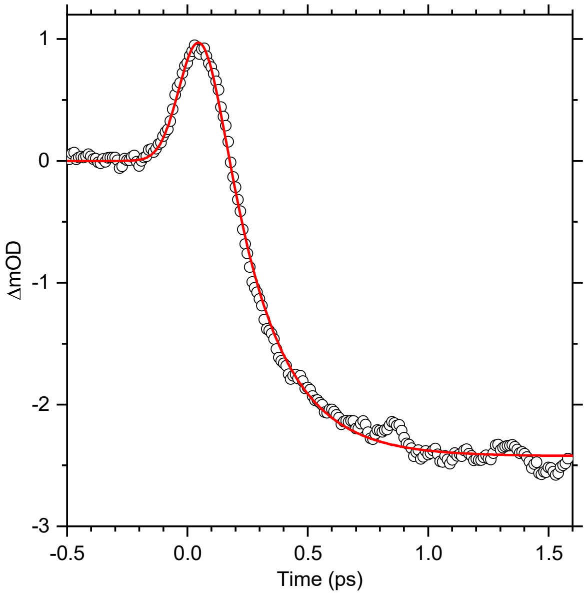

Supplementary Fig. 7 Kinetics for the decay of the S_x exciton state of c-PFBT. Open circles: Kinetics after excitation at 320 nm (averaged over the wavelength range 525–570 nm, $N_0(S_x) = 1.5 \times 10^{18}$ cm^{-3}). Solid red line: Kinetic fit (convolution with the cross correlation of 75 fs) employing a monoexponential decay and a constant negative offset. The fit provides the S_x lifetime $\tau_{x,total}$ = 206 fs.

For both excitation wavelengths, a higher initial exciton number density N_0 leads to a drastic acceleration of the decay of the bleach region, that means an accelerated recovery of the ground state S_0 due to higher-order processes. This is highlighted by the four kinetic traces shown in Supplementary Fig. 6e. This dependence on N_0 is consistent with the TrCD kinetics (Fig. 7a, main manuscript). However, interpretation of the TrCD kinetics is much easier, because it is only sensitive to the S_0 population dynamics. In contrast, for the transient absorption kinetics it is more difficult to disentangle the overlapping spectral contributions of the S_x, S_1, CP and S_0 species.

Supplementary Note 6.
Transient fluorescence experiments

Supplementary Fig. 8 shows the fluorescence decay curve of the S_1 exciton state of a c-PFBT thin film after excitation by a pulsed LED at 454 nm detected using time-correlated single photon counting (TCSPC). These measurements are at very low fluence, with an estimated initial S_1 exciton number density $N_0(S_1) = 2 \times 10^9$ cm^{-3}. This value is 9–10 orders of magnitude smaller than in the transient absorption and transient circular dichroism experiments. At such a low N_0 value, singlet–singlet annihilation processes can be completely neglected. The observed decay is therefore due to the intrinsic lifetime of the S_1 excitons. The fitting procedure provides the time constant τ_1 = 235 ps. The fit also shows a second, minor time constant τ_2 = 1070 ps (A_2 = 0.9%), which could be due to a very small amount of a fluorescing impurity in the polymer which is not detectable in the transient circular dichroism and transient absorption experiments.

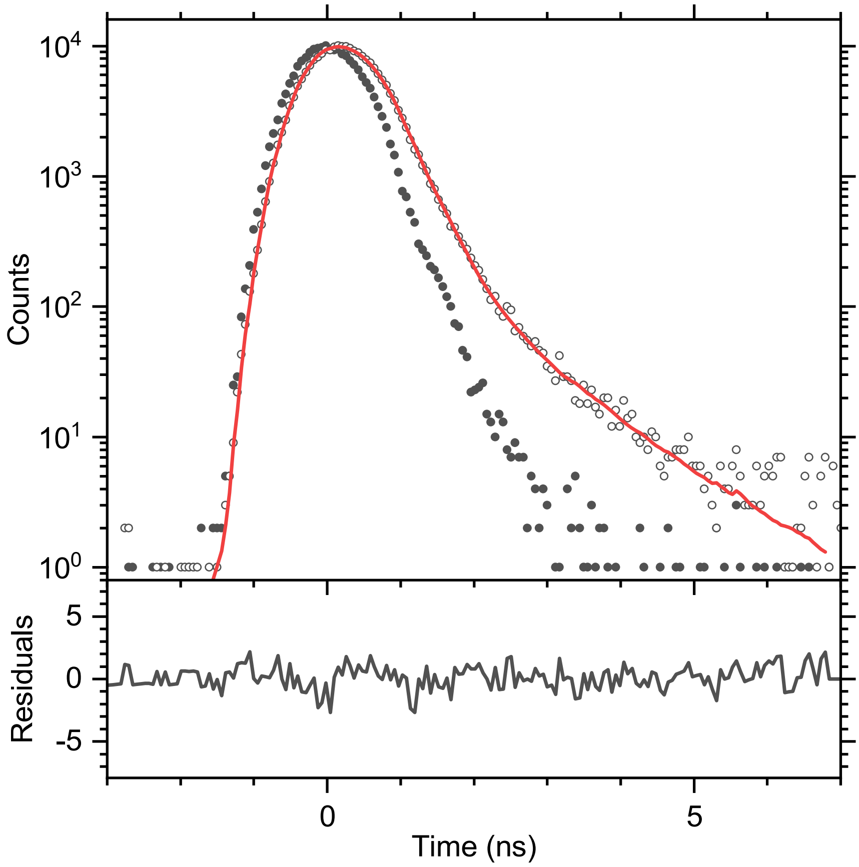

Supplementary Fig. 8 Fluorescence decay of the S_1 exciton state of a c-PFBT thin film after photoexcitation at 454 nm measured by TCSPC. Open circles: Fluorescence decay. Solid circles: Instrument response function (IRF). The red line is a biexponential fit obtained from deconvolution, with the time constants (amplitudes) τ_1 = 235 ps (A_1 = 99.1%) and τ_2 = 1070 ps (A_2 = 0.9%). Note the semilogarithmic representation. Fit residuals are provided in the bottom panel. An appropriate longpass filter (Schott GG495) was employed to cut off stray light from the excitation source.

Supplementary Note 7.
Kinetic model

To describe the relaxation processes of c-PFBT after photoexcitation, we employ the kinetic model introduced in Fig. 7b of the main manuscript, which is based on the one used by us previously for describing the photoinduced dynamics of the copolymer F8BT[1]. The mechanism is summarised and explained in the following in more detail:

$$S_x \xrightarrow{k_x} S_1 \xrightarrow{k_1} S_0 \tag{S1}$$

$$S_x \xrightarrow{k_{CPx}} e^- + h^+ \tag{S2}$$

$$2\,S_1 \xrightarrow{k_{diff}} S_n + S_0 \tag{S3}$$

$$S_1 \xrightarrow{k_{Förster}} S_0 \tag{S4a}$$

$$S_1 \xrightarrow{k_{Förster}} S_n \tag{S4b}$$

$$S_n \xrightarrow{k_n} S_1 \tag{S5}$$

$$S_n \xrightarrow{k_{CPn}} e^- + h^+ \tag{S6}$$

$$e^- + h^+ \xrightarrow{k_{rec}} S_0 \tag{S7}$$

Step S1 describes the unimolecular relaxation of the initially excited c-PFBT species to S_0. This decay starts from the S_1 singlet exciton state for the pump wavelength 450 nm (rate constant $k_1 = \tau_1^{-1}$, where τ_1 is the corresponding lifetime of 235 ps, cf. Supplementary Fig. 8) or a higher excited singlet exciton state denoted as S_x (rate constant $k_x = \tau_x^{-1}$, for excitation at 320 nm into a higher singlet absorption band), which then decays via S_1 to S_0. As discussed in our previous publication on F8BT[1], only higher electronic states, denoted as S_x, are capable of directly dissociating into a charge pair state (CP, electron (e^-) and hole (h^+)) as indicated by step S2 in the mechanism (rate constant $k_{CPx} = \tau_{CPx}^{-1}$).

Still, there are clear spectral hallmarks for the formation of the CP state upon population of S_1, as indicated by the transient absorption feature above 600 nm (Supplementary Fig. 6), and the pronounced dependence of the kinetics of the S_0 recovery on the initial exciton number density $N(S_1)$. Thus, there must be higher-order processes populating a higher-energy exciton state S_n from S_1. These are singlet–singlet annihilation (SSA) channels, which are efficient at high exciton number densities, but less important at low exciton number densities. There are two competing SSA processes: One is the diffusive bimolecular SSA channel (step S3), in which two S_1 exciton species diffuse along the polymer chains with a diffusion constant D and then react, once they reach the critical contact distance R_c. This leads to the formation of a high-energy S_n exciton state and an S_0 ground state. The corresponding rate constant k_{diff} (= τ_{diff}^{-1}) is[2]:

$$k_{diff} = 4\pi D R_c \tag{S8}$$

SSA between two S_1 excitons may also occur via nonradiative Förster resonance energy transfer (FRET)[3,4]. This requires overlap of the emission band of the S_1 donor and the ESA band of the S_1 acceptor[5]. The loss of S_1 species via FRET is of first order (step S4a, with the rate constant $k_{Förster}$ =

$\tau_{\text{Förster}}^{-1}$) and leads to simultaneous excitation of the S_1 acceptor, which is also of first order (step S4b, again with the rate constant $k_{\text{Förster}} = \tau_{\text{Förster}}^{-1}$). The total rate of S_1 loss due to FRET is therefore:

$$\frac{dN(S_1)}{dt} = -2k_{\text{Förster}} \cdot N(S_1) \tag{S9}$$

The rate constant $k_{\text{Förster}}$ displays a characteristic R^{-6} dependence on the donor–acceptor distance R, where τ_1 is the radiative S_1 lifetime of the donor and R_0 is the distance, at which there is equal probability for the radiative decay and the FRET process[3,4]:

$$k_{\text{Förster}} = \frac{1}{\tau_1}\left(\frac{R_0}{R}\right)^6 \tag{S10}$$

Therefore, close S_1 pairs react much faster than more distant pairs. Because the S_1 exciton number density $N(S_1)$ decreases during relaxation, at later times only more distant pairs with smaller $k_{\text{Förster}}$ values remain. This makes the Förster process intrinsically time-dependent, and the average rate constant $\langle k_{\text{Förster}}\rangle$ for the S_1 ensemble decreases steadily over time. In a kinetic mechanism, one would like to get rid of this R dependence and retain only the dependence on the exciton number density $N(S_1)$. Therefore, similar to earlier treatments[1,5], we use the relation between the S_1 exciton number density and the average exciton radius $\langle r\rangle$ (or, equivalently, the corresponding exciton distance $\langle R\rangle = 2\cdot\langle r\rangle$):

$$N(S_1) = \left(\frac{4}{3}\pi\langle r\rangle^3\right)^{-1} = \left(\frac{1}{6}\pi\langle R\rangle^3\right)^{-1} \tag{S11}$$

Rearranging Eq. S11 for $\langle R\rangle$ and substituting it into Eq. S10 provides (rewritten as an ensemble average)

$$\langle k_{\text{Förster}}\rangle = \frac{1}{\tau_1}\left(\frac{R_0}{\langle R\rangle}\right)^6 = \frac{\pi^2 R_0^{\,6}}{36\tau_1} N(S_1)^2 \tag{S12}$$

Reformulating Eq. S9 as an ensemble average and combining it with Eq. S12 leads to

$$\frac{dN(S_1)}{dt} = -2\langle k_{\text{Förster}}\rangle N(S_1) = -\frac{\pi^2 R_0^{\,6}}{18\tau_1} N(S_1)^3 = -k_{\text{FRET}}\, N(S_1)^3 \tag{S13}$$

As a result, the rate of the S_1 exciton loss is proportional to $N(S_1)^3$, resulting in an apparent third-order behaviour with the rate constant k_{FRET}. For the sake of an easier treatment, we therefore replace the Förster-type SSA processes S4a and S4b in the kinetic scheme by the equivalent steps S14a and S14b:

$$3\,S_1 \xrightarrow{k_F} 2\,S_n + S_0 \tag{S14a}$$

$$3\,S_1 \xrightarrow{k_F} S_n + 2\,S_0 \tag{S14b}$$

We need to use two third-order steps with the same rate constant k_F to correctly account for the stoichiometry of the FRET process. For the decay of S_1 we obtain from steps S14a and S14b

$$\frac{dN(S_1)}{dt} = -6k_F\, N(S_1)^3 \tag{S15}$$

with the third-order rate constant $k_F = k_{\text{FRET}}/6$.

To obtain the corresponding Förster radius R_0, we us a connection between $\langle k_{\text{Förster}}\rangle$ (first order, Eq. S10) and $k_{\text{FRET}} = 6\,k_F$ (Eqs. S13 and S15) by comparing the respective half-lives. The third-order half-life $t_{1/2,3.o.}$ obtained from Eq. S15 is:

$$t_{1/2,3.o.} = \frac{1}{4k_F N(S_1)^2} \tag{S16}$$

Correspondingly, we obtain from the first-order expression in Eq. S13

$$t_{1/2,1.o.} = \frac{\ln(2)}{2\langle k_{\text{Förster}} \rangle} \tag{S17}$$

Next, we use Eqs. S12, S16 and S17 to arrive at an expression for the Förster radius R_0 (under the assumption that the two half-lives should be the same):

$$R_0 = \sqrt[6]{\frac{\langle k_{\text{Förster}} \rangle 36\,\tau_1}{\pi^2 N(S_1)^2}} = \sqrt[6]{\frac{\ln(2)\,18\,\tau_1}{t_{1/2,1.o.}\,\pi^2 N(S_1)^2}} = \sqrt[6]{\frac{k_F \ln(2)\,72\,\tau_1}{\pi^2}} \tag{S18}$$

Equation S18 was employed to convert the third-order rate constant k_F (obtained from the kinetic modelling) into the corresponding R_0 value.

The remaining steps in the kinetic mechanism deal with the fate of the excited species S_n resulting from the SSA processes. The S_n exciton state either decays back to S_1 with $k_n = \tau_n^{-1}$ (step S5), or produces a charge pair (electron–hole pair, step S6) with $k_{CPn} = \tau_{CPn}^{-1}$. Recombination of the charge pair occurs on much longer time scales ($k_{rec} = \tau_{rec}^{-1}$), as indicated by step S7.

Supplementary Note 8.
Summary of optimised fit parameters for the TrCD kinetics

Supplementary Table 1 summarises the kinetic fit parameters employed for the optimised kinetic simulations of Fig. 7c in the main manuscript. Note that the dominant SSA process is the diffusive channel and that Förster resonance energy transfer plays a minor role and leads only to a small improvement of the fit.

Supplementary Table 1 Summary of parameters obtained from the kinetic analysis of the fluence-dependent measurements of c-PFBT upon photoexcitation at 320 nm.

Physical quantity (unit)	Value	Physical quantity (unit)	Value
$N_{0,\mathrm{high}}(\mathrm{S_x})$ ($\mathrm{cm^{-3}}$)	9.60×10^{18}		
$N_{0,\mathrm{low}}(\mathrm{S_x})$ ($\mathrm{cm^{-3}}$)	2.29×10^{18}		
$k_\mathrm{x}(\mathrm{S_x} \rightarrow \mathrm{S_1})$ ($\mathrm{s^{-1}}$)	4.40×10^{12}	τ_x (fs)	227
$k_\mathrm{CPx}(\mathrm{S_x} \rightarrow \mathrm{e^-} + \mathrm{h^+})$ ($\mathrm{s^{-1}}$)	4.50×10^{11}	τ_CPx (ps)	2.22
$k_\mathrm{x,total} = k_\mathrm{x} + k_\mathrm{CPx}$ ($\mathrm{s^{-1}}$)	4.85×10^{12}	$\tau_\mathrm{x,total}$ (fs)	206
		Φ_CPx (%)	9.3
$k_1(\mathrm{S_1} \rightarrow \mathrm{S_0})$ ($\mathrm{s^{-1}}$)	4.26×10^{9}	τ_1 (ps)	235
$k_\mathrm{F}(3\ \mathrm{S_1} \rightarrow \mathrm{S_n} + 2\ \mathrm{S_0})$ ($\mathrm{cm^6\ s^{-1}}$)	2.00×10^{-27}		
$k_\mathrm{F}(3\ \mathrm{S_1} \rightarrow 2\ \mathrm{S_n} + \mathrm{S_0})$ ($\mathrm{cm^6\ s^{-1}}$)	2.00×10^{-27}		
k_FRET ($\mathrm{cm^6\ s^{-1}}$)	1.20×10^{-26}		
$\langle k_\mathrm{Förster} \rangle(\mathrm{S_1} \rightarrow \mathrm{S_0})$ ($\mathrm{s^{-1}}$)[a]	1.63×10^{13} ($N_{0,\mathrm{high}}$)	R_0 (nm)[b]	11.6
	9.26×10^{11} ($N_{0,\mathrm{low}}$)		
$k_\mathrm{diff}(2\ \mathrm{S_1} \rightarrow \mathrm{S_n} + \mathrm{S_0})$ ($\mathrm{cm^3\ s^{-1}}$)	8.00×10^{-8}		
$k_\mathrm{n}(\mathrm{S_n} \rightarrow \mathrm{S_1})$ ($\mathrm{s^{-1}}$)	3.8×10^{14}	τ_n (fs)	3
$k_\mathrm{CPn}(\mathrm{S_n} \rightarrow \mathrm{e^-} + \mathrm{h^+})$ ($\mathrm{s^{-1}}$)	2.3×10^{13}	τ_CPn (fs)	40
$k_\mathrm{n,total} = k_\mathrm{n} + k_\mathrm{CPn}$ ($\mathrm{s^{-1}}$)	4.0×10^{14}	$\tau_\mathrm{n,total}$ (fs)	3
		Φ_CPn (%)	6
$k_\mathrm{rec}(\mathrm{e^-} + \mathrm{h^+} \rightarrow \mathrm{S_0})$ ($\mathrm{cm^3\ s^{-1}}$)	7.9×10^{-10}		

[a] From Eq. S12.
[b] From Eq. S18.

Supplementary Note 9.
Kinetic simulations for two limiting cases

To address the relative importance of the different singlet–singlet annihilation processes in S_1, i.e. bimolecular diffusive and FRET, we provide here two instructive results from the kinetic modelling. Supplementary Fig. 9 shows the result, which is obtained when there is only the diffusive SSA process (with optimised rate constant $k_{diff} = 1.0 \times 10^{-7}$ cm^{-3} s^{-1} and no FRET ($k_F = 0$)). The fit curves for high and low initial exciton number densities $N_0(S_x)$ are almost as good as in Fig. 7c of the main manuscript, only at longer times they are slightly too fast.

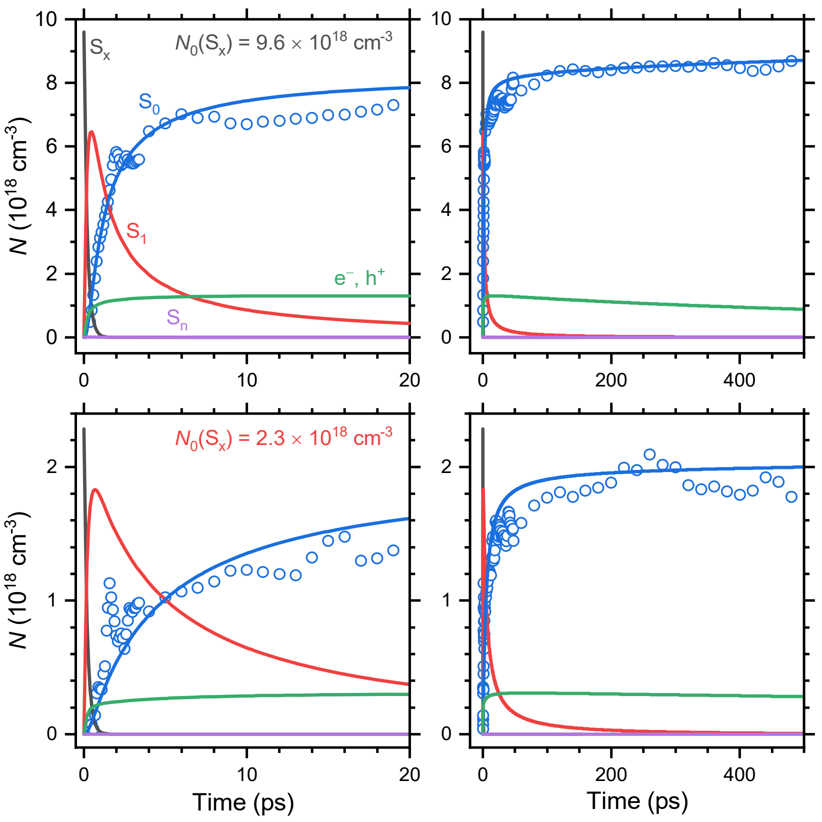

Supplementary Fig. 9 Results of the kinetic modelling with only bimolecular diffusive singlet–singlet annihilation. The model employed the same parameters as in Supplementary Table 1, with the following two exceptions: $k_{diff} = 1.0 \times 10^{-7}$ cm^3 s^{-1} and $k_F(3\ S_1 \rightarrow S_n + 2\ S_0) = k_F(3\ S_1 \rightarrow 2\ S_n + S_0) = 0$. Initial S_x exciton number densities 9.6×10^{18} cm^{-3} (top) and 2.3×10^{18} cm^{-3} (bottom). The dynamics on short time scales (up to 20 ps) are shown on the left side and long time scales (up to 500 ps) on the right side. Blue circles: Recovery of S_0 population as experimentally determined in the TrCD measurements with corresponding blue fit lines obtained from the kinetic model; black, red, green and violet lines: number density of the S_x excitons, S_1 excitons, electron-hole pairs (e^-, h^+) and the S_n excitons, as obtained from the kinetic model.

As a comparison, Supplementary Fig. 10 shows results for the other limiting case. Here, the diffusive SSA process was switched off ($k_{diff} = 0$) and the rate constant for Förster resonance energy transfer was adjusted. Importantly, it is only possible to match the rise for one of the initial exciton number density conditions. In the current example we did this for $N_0(S_x) = 9.6 \times 10^{18}$ cm^{-3} (top) using $k_F = 6.0 \times 10^{-27}$ cm^6 s^{-1}. This provides a very good match, however the simulation for the lower exciton number density $N_0(S_x) = 2.3 \times 10^{18}$ cm^{-3} (bottom) rises too slowly. This can be easily understood as

follows: The FRET process shows an apparent third-order behaviour (cf. Eq. S13) for which the half-live of the S_1 decay is proportional to $N(S_1)^{-2}$ (cf. Eq. S16). In contrast, for the second-order diffusive mechanism the half-life is proportional to $N(S_1)^{-1}$. Therefore, if the diffusive SSA process is able to match both kinetics at high and low $N_0(S_x)$ (and correspondingly $N(S_1)$) already quite well, the third-order FRET process must become much too slow with decreasing $N(S_1)$, because of the larger and larger half-life due to the inverse square dependence on $N(S_1)$. These two limiting cases from the kinetic modelling therefore clearly show that the diffusive SSA process must be the dominant process which is responsible for the observed dependence of the TrCD kinetics on the exciton number density. Adding a small contribution of the Förster mechanism slows down the kinetics somewhat at longer times, which results in the slightly better fit presented in the main manuscript (Fig. 7c).

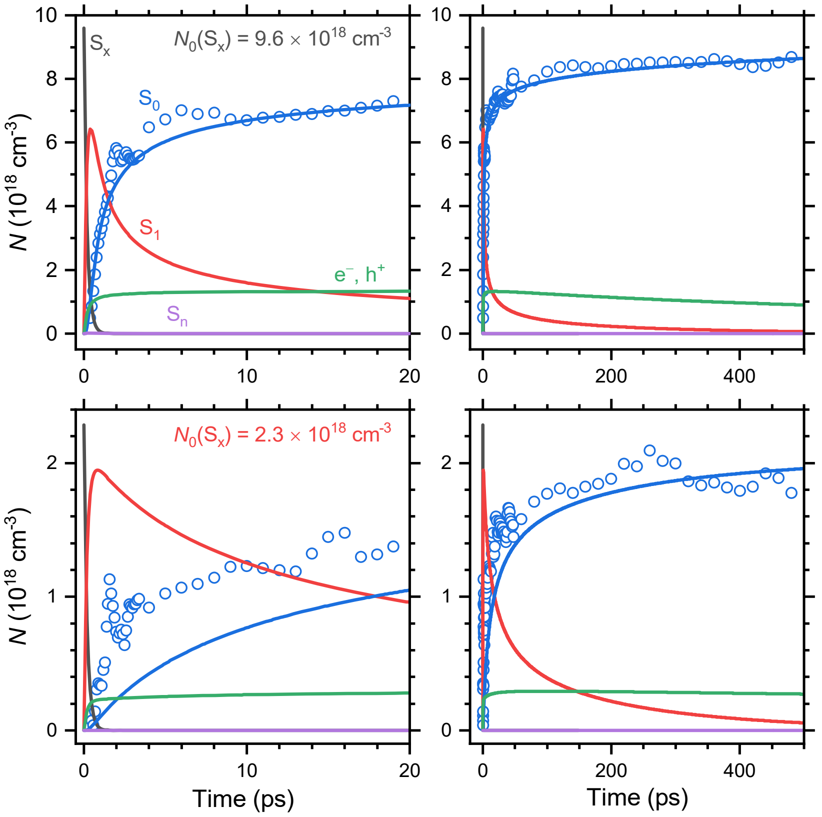

Supplementary Fig. 10 Results of the kinetic modelling with Förster resonance energy transfer (FRET) as the only singlet–singlet annihilation process. The model employed the same parameters as in Supplementary Table 1, with the following two exceptions: $k_{diff} = 0$ and $k_F(3\ S_1 \rightarrow S_n + 2\ S_0) = k_F(3\ S_1 \rightarrow 2\ S_n + S_0) = 6.0 \times 10^{-27}\ cm^6\ s^{-1}$. Initial S_x exciton number densities $9.6 \times 10^{18}\ cm^{-3}$ (top) and $2.3 \times 10^{18}\ cm^{-3}$ (bottom). The dynamics on short time scales (up to 20 ps) are shown on the left side and long time scales (up to 500 ps) on the right side. Blue circles: Recovery of S_0 population as experimentally determined in the TrCD measurements with corresponding blue fit lines obtained from the kinetic model; black, red, green and violet lines: number density of the S_x excitons, S_1 excitons, electron-hole pairs (e^-, h^+) and the S_n excitons, as obtained from the kinetic model.

A larger number of parameters enters the kinetic model (Supplementary Table 1), so we would like to finally comment on the uniqueness of the fit and possible concerns regarding overfitting the TrCD data. We note that the majority of parameters is either determined independently (and therefore can be kept fixed) or is very well defined, as summarised by the following points:

1) Because the TrCD kinetics is only sensitive to the concentration of the S_0 population (a single species), there are no ambiguities regarding relative contributions of different overlapping CD-active species with different species-associated CD values. The TrCD kinetics can be therefore taken as a direct measure of the time-dependent change of the S_0 number density.
2) We employed three different initial exciton number densities. In addition to the two initial number densities $N_{0,\mathrm{low}}(S_x)$ and $N_{0,\mathrm{high}}(S_x)$ in the TrCD experiments, there is also the condition of very low number density covered by the TCSPC experiment (Supplementary Fig. 8) which directly provides the rate constant k_1 for the decay of the S_1 state (in the absence of singlet–singlet annihilation processes), see step S1. k_1 is therefore fixed.
3) The initial exciton number densities of the TrCD experiments and the TCSPC experiment were independently determined and are therefore also fixed.
4) In addition, the transient absorption kinetics in Supplementary Fig. 7 provides the total rate constant $k_{x,\mathrm{total}}$ for the decay of the initially populated S_x state, which decays via two parallel channels. This value poses a strict limit on the sum of the two rate constants for the formation of the S_1 state (k_x) and the formation of the charge pair state (k_{CPx}), i.e. $k_{x,\mathrm{total}} = k_x + k_{CPx}$.
5) The ratio of the rate constants k_x and k_{CPx} (steps S1 and S2) and the ratio of the rate constants k_n and k_{CPn} (steps S5 and S6) are also well defined by the experimentally observed yield for the long-lived CP state, which can be directly determined from the incomplete recovery of the TrCD kinetics. Only 85% of the S_0 population is recovered at about 500 ps, see Supplementary Figs. 9 and 10 (top right panel, blue line), so only 15% of the initially excited c-PFBT population ends up in the CP state (green line). The rate constant $k_{x,\mathrm{total}}$ was measured independently. Therefore, k_x and k_{CPx} are well defined, with $k_x >> k_{CPx}$. k_n and k_{CPn} are also well defined: Because the total yield of the CP state is only 15%, k_n must be considerably larger than k_{CPn}. In addition, the sum $k_{n,\mathrm{total}} = k_n + k_{CPn}$ must be large, because the singlet–singlet annihilation processes would otherwise be too slow, and it would not be possible to fit the fast rise of the TrCD signal at early times.
6) As demonstrated by the systematic parameter variations in Supplementary Figs. 9 and 10, the dependence of the S_0 recovery on the different initial exciton number densities is governed by the competition of bimolecular diffusive SSA (step S3) and FRET (steps S14a and S14b), which leads to accurate values for the SSA rate constants k_{diff} and k_F.
7) The final electron–hole recombination step S7 has only a very weak influence on time scales up to 500 ps, as shown by the green lines in Supplementary Figs. 9 and 10 (right panels), and therefore the value for k_{rec} should only be taken as a rough estimate.

We therefore conclude that all parameters entering the kinetic model are well defined and provide detailed kinetic information regarding the individual processes involved.

Supplementary References

1. Morgenroth, M., Scholz, M., Lenzer, T. & Oum, K. Ultrafast UV-vis transient absorption and circular dichroism spectroscopy of a polyfluorene copolymer showing large chiral induction. *J. Phys. Chem. C* **124**, 10192-10200 (2020).
2. Powell, R. C. & Soos, Z. G. Singlet exciton energy transfer in organic solids. *J. Lumin.* **11**, 1-45 (1975).
3. Förster, T. Zwischenmolekulare Energiewanderung und Fluoreszenz. *Ann. Phys. (Berlin, Ger.)* **437**, 55-75 (1948).
4. Förster, T. Transfer mechanisms of electronic excitation. *Discuss. Faraday Soc.* **27**, 7-17 (1959).
5. Stevens, M. A., Silva, C., Russell, D. M. & Friend, R. H. Exciton dissociation mechanisms in the polymeric semiconductors poly(9,9-dioctylfluorene) and poly(9,9-dioctylfluorene-co-benzothiadiazole). *Phys. Rev. B* **63**, 165213 (2001).
